# A phase II trial of S-1 and oxaliplatin in patients with advanced hepatocellular carcinoma

**DOI:** 10.1186/s12885-018-4039-9

**Published:** 2018-03-05

**Authors:** Dae-Won Lee, Kyung-Hun Lee, Hee-Jun Kim, Tae-Yong Kim, Jin-Soo Kim, Sae-Won Han, Do-Youn Oh, Jee Hyun Kim, Seock-Ah Im, Tae-You Kim

**Affiliations:** 1 Department of Internal Medicine, Seoul National University Hospital, 101 Daehang-ro, Jongno-gu, Seoul, 110-744 South Korea; 2 Cancer Research Institute, Seoul National University College of Medicine, Seoul, South Korea; 3 Department of Internal Medicine, Chung-Ang University College of Medicine, Seoul, South Korea; 4 Department of Internal Medicine, Seoul Metropolitan Government Seoul National University Boramae Medical Center, Seoul, South Korea; 5 Department of Internal Medicine, Seoul National University Bundang Hospital, Seongnam, South Korea

**Keywords:** Hepatocellular carcinoma, Chemotherapy, Phase II, Oxaliplatin, S-1

## Abstract

**Background:**

Oxaliplatin is a platinum derivative that has shown efficacy in advanced hepatocellular carcinoma. S-1 is an oral fluoropyrimidine that has substituted for 5-fluorouracil in many cancers. This was a multicenter, open-label, single-arm phase II trial that evaluated the efficacy of S-1 and oxaliplatin (SOX) in advanced hepatocellular carcinoma. All patients included in the present study were systemic treatment-naïve. Prior treatment with sorafenib was allowed, but other treatments were not.

**Methods:**

Patients received S-1 (40 mg/m^2^ twice daily from day 1–14) and oxaliplatin (130 mg/m^2^ on day 1) every 3 weeks. The primary end point was time to progression (TTP). Secondary end points included progression-free survival, overall survival (OS), response rate, and safety profile.

**Results:**

Thirty six patients with advanced hepatocellular carcinoma were included in this study. The median TTP was 3.0 months (95% confidence interval (CI), 0.75–5.25), and the median OS was 10.3 months (95% CI, 6.4–14.3). Bone metastasis was associated with poorer TTP and OS. The efficacy of SOX was unaffected by prior sorafenib or locoregional therapy. The objective response rate was 13.9%. No grade 4 toxicity or death from adverse events occurred. The most common grade 3 toxicities were neutropenia (13.9%), thrombocytopenia (13.9%), and diarrhea (8.3%).

**Conclusions:**

Although this trial did not meet its primary end point, the SOX regimen showed comparable efficacy and safety to the 5-fluorouracil, leucovorin, and oxaliplatin (FOLFOX) regimen. As the SOX regimen is easier for patients, SOX may be a reasonable substitute for FOLFOX in hepatocellular carcinoma.

**Trial registration:**

Clinicaltrials.gov NCT01429961. Registered 7 September 2011.

## Background

Liver cancer is the second and sixth most frequent cause of death from cancer in men and women, respectively [1]. Overall, 70% to 90% of liver cancers are hepatocellular carcinoma, which has poorer prognosis, as most patients are diagnosed at advanced stages and have underlying hepatic dysfunction [2]. Sorafenib, a multi-tyrosine kinase inhibitor, has shown efficacy in advanced hepatocellular carcinoma and is the only molecular targeted agent approved for hepatocellular carcinoma based on two phase III trials [3, 4].

Hepatocellular carcinoma is highly refractory to conventional systemic chemotherapy [5]. Although doxorubicin is a palliative treatment in hepatocellular carcinoma, no studies have found strong evidence for the survival benefit of doxorubicin. However, in a phase III clinical trial performed in Asia (EACH trial) which compared the efficacy of FOLFOX (5-fluorouracil (5-FU), leucovorin, and oxaliplatin) and doxorubicin, FOLFOX showed prolonged progression free survival (PFS, 2.93 months vs. 1.77 months) and overall survival (OS, 6.40 months vs. 4.97 months) compared to doxorubicin [6]. In a study performed in a Western population, gemcitabine combined with oxaliplatin showed efficacy (median PFS and OS of 4.5 and 11.0 months, respectively) in a multicenter retrospective study [7]. Although conventional chemotherapy has never been compared directly with sorafenib, these findings show that conventional chemotherapy may be an option for advanced hepatocellular carcinoma patients.

S-1 is an oral fluoropyrimidine agent, consisting of tegafur (a pro-drug of 5-FU), gimeracil, and oteracil. Gimeracil and oteracil decrease 5-FU anti-metabolite degradation and achieve higher concentrations of 5-FU in the plasma and tumor tissues. Recently, 5-FU has been substituted by oral fluoropyrimidines, such as capecitabine or S-1, to treat many cancers based on phase III study results showing comparable efficacy and better safety profiles with oral fluoropyrimidines [8–12]. Therefore, we performed a phase II study to evaluate the efficacy and safety of S-1 and oxaliplatin (SOX) in patients with advanced hepatocellular carcinoma.

## Methods

### Study design and participants

This study was a multicenter, open-label, single-arm, phase 2 trial that evaluated the efficacy of SOX in advanced hepatocellular carcinoma. Patients with hepatocellular carcinoma of Barcelona Clinic Liver Cancer stage C that was either refractory or not amenable to locoregional therapy were eligible for the present study. Hepatocellular carcinoma was diagnosed based on 2005 AASLD practice guidelines [13]. Either histopathological findings from tumor tissue or non-histological diagnosis based on triphasic CT scan and/or gadolinium enhanced MRI was required [13]. Without histological confirmation, liver mass ≥ 2 cm with characteristic vascularization (either on a triphasic CT scan or gadolinium-enhanced MRI) or AFP ≥ 200 μg/L was required. In patients with a 1 to 2 cm liver mass, characteristic vascularization on both a triphasic CT scan and gadolinium-enhanced MRI were required with concomitant liver cirrhosis. At least one measurable extrahepatic lesion based on the Response Evaluation Criteria in Solid Tumors (RECIST) criteria 1.1 was required. Other main inclusion criteria were an age of over 18 years; Eastern Cooperative Oncology Group performance status (ECOG PS) of 0 to 2; Child-Pugh class A; adequate bone marrow, hepatic, and renal function [absolute neutrophil count (ANC) ≥ 1500/μL; platelet count ≥100,000/μL, total bilirubin ≤2 X upper limit of normal (ULN); serum transaminases ≤2.5 X ULN; alkaline phosphatase ≤2.5 X ULN; serum creatinine ≤1.5 X ULN]. Patients with serum transaminases and alkaline phosphatase ≤5 X ULN could be included if they had a normal total bilirubin level. Patients were excluded if they had either previous systemic chemotherapy (except prior sorafenib) or history of another malignancy within the last 5 years.

The study protocol was reviewed and approved by the institutional review board of Seoul National University Hospital, Seoul, Korea [H-1010-054-336]. This study was conducted in accordance with the recommendations of the Declaration of Helsinki for biomedical research involving human subjects and the Guidelines for Good Clinical Practice (ClinicalTrial.gov Identifier: NCT01429961). Written informed consent was obtained from each patient before enrollment.

### Treatment and dose modification

S-1 was administered orally at a dose of 40 mg/m^2^ twice daily for 14 days (80 mg/m^2^/day), followed by a 7-day rest period. Oxaliplatin was given as a 120–minute infusion on day 1 of each cycle at a dose of 130 mg/m^2^. Dose reduction of S-1 (30 mg/m^2^ twice daily, which is 60 mg/m^2^/day) and Oxaliplatin (100 mg/m^2^) was allowed per the discretion of the treating physician, as most patients with HCC have compensated liver cirrhosis despite a Child-Pugh class A score. Treatment was repeated every 3 weeks until disease progression, unacceptable toxicity, or withdrawal of patient consent. Drug administration was delayed until an ANC of ≥1500/μL and platelet counts of ≥100,000/μL, and recovery from non-hematological toxicity to baseline or less than or equal to grade 1. S-1 was reduced to a dose of 30 mg/m^2^ twice daily (60 mg/m^2^/day) and oxaliplatin was reduced to a dose of 100 mg/m^2^ on all subsequent cycles for febrile neutropenia, grade 4 neutropenia, grade 3/4 thrombocytopenia, or greater than or equal to grade 3 non-hematological toxic effects.

### Assessment

Baseline assessments included medical history, physical examination, electrocardiography, chest X-rays, abdominal and pelvic CT scans (gadolinium enhanced MRI when necessary), complete blood counts, serum electrolytes and chemistry, and urine analysis. Tumor response was assessed using RECIST criteria 1.1, with contrast-enhanced triphasic CT scans at baseline and every two cycles (6 weeks). Toxicity was evaluated at each cycle per the National Cancer Institute Common Terminology Criteria for Adverse Events, version 3.0.

### Statistical analysis

The primary end point of this study was time to progression (TTP), which was defined as the time from study enrollment to tumor progression. Deaths without progressive disease were censored in the TTP analysis. Secondary end points were OS, PFS, response rate, and toxicity. OS was calculated from the date of study enrollment to the date of death. PFS was defined as the interval between the date of study enrollment and first date of documented progressive disease or the date of death from any cause. Tumor response was assessed using RECIST criteria 1.1. TTP, PFS, and OS were estimated using the Kaplan-Meier method and comparisons were made using log-rank tests. Statistical analysis was performed using SPSS software for Windows, version 18.0 (SPSS, Chicago, IL, USA).

We hypothesized an increased TTP from 2.8 months to 4.0 months in the SOX group. With an alpha of 0.10 and a power of 80%, 34 patients were required for this study. Considering a 10% loss to follow-up rate, the target enrollment was 38 patients.

## Results

### Patient characteristics

Between May 27, 2011, and August 28, 2014, 36 patients were enrolled. All patients included in our cohort met the diagnostic criteria for both 2005 and 2010 AASLD guidelines [14]. Baseline characteristics of the patients are summarized in Table 1. The median patient age was 58 years (range, 21–74), and 33 patients (91.7%) were male. All patients had at least 1 extrahepatic measurable lesion and good ECOG PS. Per the inclusion criteria, all patients were classified as Child-Pugh class A and had adequate hepatic function. On the baseline CT/MRI, 94.4% (34/36) showed liver cirrhosis. Of the 36 patients, 31 patients were treated with prior sorafenib or locoregional therapy (TACE, PEIT, or RFA). One patient did not receive either prior sorafenib or locoregional therapy.

**Table 1:** Patient characteristics

	Number of patients (%) (*N* = 36)
Age	
Median (range)	58 (21–74)
≥ 65 years	10 (27.8%)
Sex	
Male	33 (91.7%)
Female	3 (8.3%)
Hepatocellular carcinoma etiology	
Hepatitis B	26 (72.2%)
Hepatitis C	1 (2.8%)
Alcoholic cirrhosis	8 (22.2%)
Other	1(2.8%)
Metastastic sites	
Lung	22 (61.1%)
Lymph node	11 (30.6%)
Bone	9 (25.0%)
Other	11 (30.6%)
ECOG PS	
0	23 (63.9%)
1	13 (36.1%)
2	0 (0.0%)
Any prior therapy	
Sorafenib	31 (86.1%)
Locoregional therapy^a^	31 (86.1%)
AFP	
< 200 ng/mL	22 (61.1%)
≥ 200 ng/mL	14 (38.9%)
PIVKA	
< 400 mAU/mL	17 (47.2%)
≥ 400 mAU/mL	19 (52.8%)

Abbreviations: *ECOG*, Eastern Cooperative Oncology Group, *PS* performance status

^a^includes transcatheter arterial chemoembolization (TACE), percutaneous ethanol injection therapy (PEIT), and radiofrequency ablation (RFA)

### Efficacy

Response evaluation was available for all 36 patients. One patient achieved a complete response (CR, 2.8%), 4 had a partial response (PR, 11.1%), 13 had stable disease (SD, 36.1%), and 18 had progressive disease (PD, 50.0%). The overall response rate (ORR) was 13.9% and the disease control rate (DCR) was 50.0%.

**Fig. 1:**
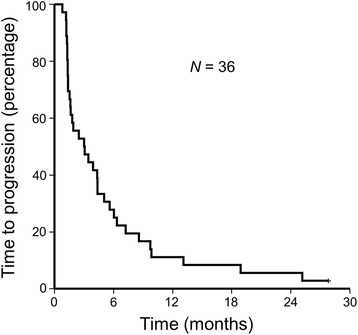
Kaplan–Meier survival curve for time to progression

**Fig. 2:**
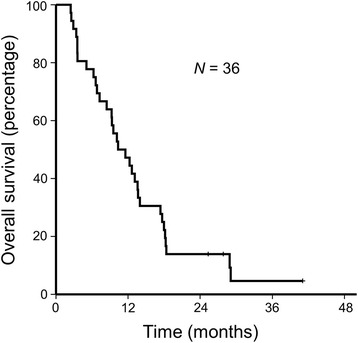
Kaplan–Meier survival curve for overall survival

**Fig. 3:**
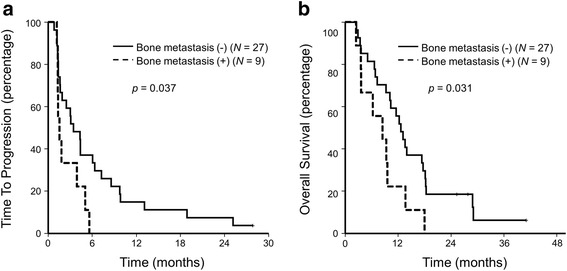
Kaplan–Meier curves of TTP and OS according to the presence of bone metastasis. Abbreviations: TTP, time to progression; OS, overall survival

With a median follow-up of 10.9 months, 35 progression events and 34 deaths occurred. PFS and TTP were the same in our cohort as no patients died without progression. The median TTP and PFS were 3.0 months (95% confidence interval (CI), 0.7–5.3) (Fig. 1), and the median OS was 10.3 months (95% CI, 6.4–14.3) (Fig. 2). The trial did not meet its primary end point of a 4-month TTP. Bone metastasis was associated with poorer survival (hazard ratio (HR) for TTP 2.31, 95% CI 1.02–5.21, *p* = 0.045) (HR for OS 2.36, 95% CI 1.05–5.30, *p* = 0.037) (Fig. 3). Gender, age, PS, AFP level, PIVKA level, prior sorafenib treatment, prior locoregional therapy, and lung or liver metastasis were unassociated with TTP and OS. In patients treated with prior sorafenib (31/36, 86.6%), median TTP and OS were 3.0 (95% CI, 1.2–4.8) months and 9.5 (95% CI, 5.4–13.7) months, respectively.

### Toxicity

Patients received a total of 199 treatment cycles, with a median of 3 cycles (range, 1–27) per patient. Study treatments were discontinued in 2 patients (5.5%) because of severe adverse events, including spontaneous bacterial peritonitis and hepatocellular carcinoma rupture. Nine patients (25.0%) started chemotherapy at a reduced dose and 27 (75.0%) started chemotherapy at the regular dose. Of the 27 patients who started chemotherapy at the regular dose, 11 (40.7%) had their dose reduced during the chemotherapy cycle due to toxicity. This includes four cases of grade 3 thrombocytopenia, three cases of grade 3 diarrhea, one case of grade 3 neutropenia, one case of grade 3 sensory neuropathy, one case of grade 2 gastrointestinal bleeding, and one case of septic shock due to pneumonia. All 11 patients recovered from the adverse event and received subsequent chemotherapy at a reduced dose. No treatment-related death or grade 4 toxicity occurred during the study. Detailed toxic events per patient are shown in Table 2. The most common adverse event of any grade was sensory neuropathy (52.8%), followed by thrombocytopenia (41.7%), diarrhea (38.9%), nausea (36.1%), anorexia (33.3%), and neutropenia (30.1%). The most common grade 3 toxicities were neutropenia (13.9%), thrombocytopenia (13.9%), and diarrhea (8.3%).

**Table 2:** Toxicity profile

	All courses (total *N* = 36)			
	Grade 1	Grade 2	Grade 3	Total
Neutropenia^a^	N/A	6 (16.7%)	5 (13.9%)	11 (30.1%)
Thrombocytopenia^a^	N/A	10 (27.8%)	5 (13.9%)	15 (41.7%)
Bilirubin^a^	N/A	2 (5.6%)	0 (0.0%)	2 (5.6%)
AST/ALT^a^	N/A	1 (2.8%)	1 (2.8%)	2 (5.6%)
Asthenia	4 (11.1%)	3 (8.3%)	1 (2.8%)	8 (22.2%)
Anorexia	10 (27.8%)	2 (5.6%)	0 (0.0%)	12 (33.3%)
Nausea	13 (36.1%)	0 (0.0%)	0 (0.0%)	13 (36.1%)
Vomit	5 (13.9%)	0 (0.0%)	0 (0.0%)	5 (13.9%)
Abdominal pain	7 (19.4%)	1 (2.8%)	0 (0.0%)	8 (22.2%)
Stomatitis	8 (22.2%)	1 (2.8%)	0 (0.0%)	9 (25%)
Diarrhea	8 (22.2%)	3 (8.3%)	3 (8.3%)	14 (38.9%)
Constipation	5 (13.9%)	2 (5.6%)	0 (0.0%)	7 (19.4%)
Sensory neuropathy	14 (38.9%)	4 (11.1%)	1 (2.8%)	19 (52.8%)
Motor neuropathy	0 (0.0%)	1 (2.8%)	0 (0.0%)	1 (2.8%)
Skin rash	2 (5.6%)	1 (2.8%)	0 (0.0%)	3 (8.3%)

Abbreviations: *AST* Aspartate aminotransferase, *ALT* Alanine aminotransferase, *N/A* not assessed

^a^Toxicities with Grade 2 or more were counted for these items

## Discussion

The results of our phase 2 study show that the SOX regimen may be an option for managing patients with advanced hepatocellular carcinoma. The median TTP/PFS was 3.0 months (95% CI, 0.7–5.3), median OS was 10.3 months (95% CI, 6.4–14.3), ORR was 13.9%, and DCR was 50.0%. Although the study did not meet its primary end point (TTP of 4.0 months), SOX showed comparable efficacy with FOLFOX in the EACH trial (PFS: 2.93 months, OS: 6.40 months, ORR: 8.15%, DCR: 52.17%) [6].

In this study, no grade 4 toxicity resulted from the SOX regimen. Most adverse events were grade 1/2 and common grade 3 toxicities were neutropenia and thrombocytopenia. However, most patients with grade 3 toxicity were easily managed and tolerated after a dose reduction. Although 2 patients stopped chemotherapy due to severe adverse events, these events (spontaneous bacterial peritonitis and hepatocellular carcinoma rupture) were not directly associated with chemotherapy toxicity. Although we cannot directly compare our results to the EACH trial, the SOX regimen showed a better safety profile than the FOLFOX regimen [6]. Fifty-five percent of patients treated with FOLFOX had an adverse event over grade 3, and 6% died from the severe adverse event. In addition, 23% of patients discontinued FOLFOX chemotherapy due to adverse events.

Sorafenib is the current standard of care in managing advanced hepatocellular carcinoma. Several studies have investigated the efficacy of novel molecular-targeted agents (sunitinib, everolimus, brivanib, linifanib, and ramucirumab) in first-line or second-line settings [15–20]. However, all studies were negative except for regorafenib which showed efficacy in sorafenib-resistant hepatocellular carcinoma [21]. Currently, few options exist to manage patients with advanced hepatocellular carcinoma. In addition, sorafenib efficacy is modest in Asian patients compared to Western patients [3, 4]. Although FOLFOX has not been compared directly with sorafenib, previous results support FOLFOX as a reasonable option for managing advanced hepatocellular carcinoma. In this study we evaluated the efficacy of SOX in advanced hepatocellular carcinoma. S-1 is an oral fluoropyrimidine which can substitute 5-FU in many cancers. Our results show that SOX is comparable to FOLFOX and may be an alternative to FOLFOX. Moreover, TTP and OS were unaffected by whether the patients received prior sorafenib or locoregional therapy. In a single center retrospective study, the SOX regimen showed efficacy comparable to that of sorafenib in advanced hepatocellular carcinoma [22]. Thus, the SOX regimen may be an effective option in pre-treated or treatment-naïve advanced hepatocellular carcinoma patients.

In this study, bone metastasis was associated with poorer survival in patients treated with the SOX regimen. As this study was a single-arm study, we cannot determine whether this poorer survival was due to its innate aggressive biology of the metastasis or its resistance to chemotherapy. Although bone is a frequent extrahepatic metastatic site, its prognostic role is unknown [23]. Evidence shows that hepatocellular carcinoma patients with bone metastasis may derive survival benefit from locoregional and/or systemic chemotherapy compared to the best supportive care (OS, 9.7 vs. 2.9 months, log-rank test *p* = 0.081) [24]. Prognostic and predictive roles of bone metastasis in patients with hepatocellular carcinoma should be assessed in larger studies.

## Conclusion

This phase II study indicates that combination therapy with S-1 and oxaliplatin may be effective in patients with advanced hepatocellular carcinoma. Toxicity was moderate, but manageable. Patients with bone metastasis showed poorer survival following SOX treatment. As the SOX regimen shows comparable efficacy with FOLFOX and is easier for patients, SOX may be a reasonable substitute for FOLFOX in patients with advanced hepatocellular carcinoma.

## Abbreviations

5-FU: 5-fluorouracil
ANC: Absolute neutrophil count
CI: Confidence interval
CR: Complete response
DCR: Disease control rate
ECOG PS: Eastern Cooperative Oncology Group performance status
FOLFOX: 5-fluorouracil, leucovorin, and oxaliplatin
HR: Hazard ratio
ORR: Overall response rate
OS: Overall survival
PD: Progressive disease
PFS: Progression free survival
PR: Partial response
RECIST: Response Evaluation Criteria in Solid Tumors
SD: Stable disease
SOX: S-1 and oxaliplatin
TTP: Time to progression
ULN: Upper limit of normal
